# Development of task switching and post-error-slowing in children

**DOI:** 10.1186/1744-9081-5-38

**Published:** 2009-09-15

**Authors:** Rashmi Gupta, Bhoomika R Kar, Narayanan Srinivasan

**Affiliations:** 1 Centre of Behavioural and Cognitive Sciences, University of Allahabad, Allahabad, 211002, India

## Abstract

**Background:**

Executive control processes such as task switching and error processing have been shown to change with age. The present study explored from a developmental perspective whether shared or different mechanisms underlie these processes.

**Methods:**

The sample included 180 children (30 in each of the six age groups from 6-11 years) who were required to perform two different tasks: identification of a digit, or counting the number of digits. We computed switch costs as a function of response-repetition, stimulus-response (S-R) compatibility, and post-error-slowing. We also analyzed reaction time distributions.

**Results and discussion:**

The results showed a switch cost in the response-repetition condition, with a reduction in switch cost between 7 to 8 and 9 to 10 years of age, and an S-R compatibility effect in 6 to 9 years old children. Reaction time (RT) distributions showed that the decrement in the switch cost is due to the overall decrease in RTs in fast (5^th ^percentile) trials in 9 to 11 year olds, and slow (95^th ^percentile) trials in 7 to 8 and 9 to 11 years old children, in both the task switch and non-switch trials. A major reduction in RT was found between 9 to 11 years in both the response type and S-R compatibility type conditions. RT distributions for post-error trials revealed that the large decrement seen in 7 to 8 and 9 to 10 years old children is primarily due to the sudden decrease in RTs in the fast and slow trials respectively. The developmental pattern of error processing was similar to one component of task switching (switch cost of the response-repetition condition), indicating that inhibition could be a common mechanism underlying both the processes. However, the failure to maintain task set was found only with task switching.

**Conclusion:**

The development of task switching and error processing is not gradual. The developmental pattern of error processing is similar to that of the switch cost of the response-repetition condition in task switching, indicating that inhibition could be a common mechanism underlying both processes. The present results have implications for theories of executive control.

## Background

Previous research has shown age-related changes occur in executive control processes that are critical for perception and action. For example, inhibitory control develops throughout childhood and does not reach full maturity until 12 years of age or later [1,2]. A small number of studies have examined the developmental trajectory of executive control processes [3-6]. In the present study, we focus on the development of two such control processes: task switching and error processing.

Task switching is a control process that enables flexible switching between task rules and responses. In the task switching paradigm, people perform two tasks alternately. The time to prepare for the upcoming task is often varied by the experimenter. A mixture of fast and slow responses is found on trials that demand a task switch but provide ample time for advance preparation. Typically, the fast responses are fast on trials on which the task is repeated. The slow responses are slow on trials that demand a task switch, but provide no time for advance preparation. Possible explanations include the roles of active preparation or passive interference [3].

Preparation theories focus on the active preparation for task performance. These processes allow an individual to prepare in advance by reconfiguring their internal task state. In contrast, interference theories rely on passive decay of the previous task-set stored in working memory. De Jong [7] has argued that the residual switch costs can be accounted for by a failure to prepare on a subset of trials. The failure to engage hypothesis [7] states that people are capable of advance preparation when a switch between two tasks has to be made, but fail to do so on a subset of trials. Initiation of the response is quick when people successfully engage in advance preparation during the preparation interval, but when people fail to engage in advance preparation the initiation of the response is slow.

The effect of age on task switching has mainly been examined in adults. Only a few studies have examined age-related changes in the switch costs (i.e., the difference in reaction time when switching between tasks versus repeating tasks within a mixed task block) in children [3,4,8-10]. Different components of task switching can be investigated by manipulating the delay between consecutive trials, or between the task cue and the target trials. These manipulations can inform us on whether performance deficits are associated with an inability to inhibit the previous task set (i.e. overriding the previously relevant S-R rule), or with difficulty in activating the upcoming task set (i.e. rule retrieval). Developmental studies have reported that the switch costs decrease as children grow older [3], but the underlying processes of this trajectory remain unclear [9].

Cepeda and colleagues [3] have examined age-related differences in task switching performance, in terms of changes in processes responsible for preparation and interference control. They manipulated cue-target interval (CTI) and inter-trial interval/response-cue interval (ITI/RCI) and found that the benefit in increasing the CTI was similar for all the age groups. In contrast, increasing the RCI resulted in a decrease in switch costs for the young adults, but not for the children. These results indicated that age did not interact with the preparation time (CTI) and ITI for children, indicating that switching performance was not dependent on CTI/RCI. The effect of both of these variables did not change with age. They observed larger switch costs among young children with 7 to 9 years of age, but this decreased with age. Performance improved with increased preparation time for the next task. There was a reduction in improvement as the interval between the response to one task and the cue specifying the next task was increased. It is suggested that young children probably experience more interference from the previous stimulus-response (S-R) association, indicating larger carry-over effects from the previous trial [3].

Kray and Colleagues [9] examined the age-related changes in task switching in children (mean: 9.4 years), young adults (21.5 years), and older adults (65.3 years) using categorization of pictures by object or by color. The tasks were indicated by semantic instructional cues. Dibbets and Jolles [11] focused on task switching in children younger than 6 years (58-152 months old) and the development of this ability across childhood. The results indicated that the children younger than 6 years are able to switch between two tasks and that the general performance increased with age. Young children (58-89 months) displayed larger global switch costs than older children (106-156 months), i.e. they made more errors when the tasks were presented randomly, compared with the repeated task baseline. These findings suggest that the ability to maintain and manipulate two different tasks in working memory is present, but not fully developed, in young children. These results also indicate that the performance deficits in children are associated with an inability to inhibit the previous task set.

It has been argued that when a task is repeated, individuals benefit from response repetition if the response-stimulus interval is short (automatic facilitation effect) [12]. However, when a task is not repeated, individuals are hindered by response repetition (reversed repetition effect), which has been linked to an inhibitory process [13,14]. Both the effects are sensitive to developmental changes [4,15]. For example, Smulders and colleagues [15] found that automatic facilitation was larger among younger children (7 to 9 years of age), indicating larger carry-over effects from prior S-R associations. Crone and colleagues [4] found greater switch costs with young children (7 to 8 years of age) compared to adults for task switching with repeating responses. This age difference decreased with the increase in the interval between the previous response and the upcoming stimulus.

Crone and colleagues [4] also examined the influence of carried-over inhibition in tasks with different stimulus-response mapping strengths (compatible and incompatible responses). Switch costs were usually larger when individuals needed to switch to the stronger (more dominant) task than to a weaker task. Allport and colleagues [16] have argued that the extra inhibition of the stronger task set is required to enhance performance with the weaker task set, and therefore, inhibition carries over to the next trial. Crone and colleagues [4] found that switch costs were larger when switching to the compatible task than to the incompatible task, but this effect did not differ across all the age groups (7-8 years, 10-12 years, and 20-25 years). They concluded that younger children build strong transient associations between task sets and response sets, which interfere with their ability to switch to the currently intended actions.

When switching between actions, errors may occur. Online processing of such errors and making subsequent adjustments in processing is important for cognitive control. Error processing is evident in the slowing of responses following errors in speeded reaction time tasks [17] and after failed attempts to inhibit a response [17,18]. Many studies have shown that following the detection of an error, participants adjust processing speed to achieve an adequate level of accuracy [19]. Children also monitor errors, as indicated in the slowing down following errors in speeded choice reaction time tasks [20].

Error processing as indexed by post-error slowing (PES) varies with age in the age range of 7 to 16 years, with larger PES for younger compared to older children [21]. Kramer and colleagues [22] found that elderly participants showed larger PES than younger adults following non-stopped responses (50 ms vs. 21 ms). Together, these studies indicate a curvilinear pattern of development in PES over the life span. PES initially decreases with age, reaches the maximum at adulthood and increases among older adults. A similar pattern of development has also been observed for inhibitory control [23]. Posner and Rothbart [24] found that by 48 months, children were usually able to inhibit a response appropriately. In addition, they also found that the ability to detect an error (indicated by PES) seemed to develop at an earlier age than did the ability to inhibit responses.

Error processing studies with event related potentials have shown that error related negativity (ERN) amplitude (reflecting unconscious detection of an error) in error trials increased with age [5]. However, the error-positivity (*Pe*) amplitude (reflects conscious error recognition and performance adjustment after an error) did not change with age. In the case of the correct trials, most participants produced a small negativity corresponding to the timing of the ERN in the error trials. This correct-response negativity amplitude was larger with children (7 to 12 years of age) than with adults.

One important question related to cognitive control is the possibility of common mechanisms in task switching and error processing. It is well established that inhibition is an important contributor to switch costs, and an important mechanism underlying task switching [25]. However, the precise mechanisms underlying PES are still a matter of debate [21]. Possible hypotheses include automatic inhibition of the response after an erroneous trial, as well as additional comparisons between actual response and representations of intended responses [26-28]. We examined both the processes using the same (task switching) task, and argue that inhibition could be a mechanism that is common to both task switching and error processing (PES). We have defined inhibition as a control function, which is required in withholding the response and delaying the response.

In the present study, we explored from a developmental perspective the possibility of shared mechanisms underlying task switching and error processing. Specifically, we wanted to examine whether the development of these processes across age would show similarities or differences. If the underlying mechanisms in task switching and error processing are completely dissociable, then their developmental trends might be different. The developmental pattern of these control processes would also have implications for developmental disorders such as attention deficit hyperactivity disorder (ADHD) which are characterized by executive control deficits [29]. Hence, we examined task switching and error processing with the same task, enabling us to control for differences in experimental design typically associated with the different tasks used to study these processes.

Previous studies on task switching and error processing have examined these cognitive processes with a lesser number of cases, or coarse groupings of ages. For example, Crone and colleagues [4] compared 7-8 with 10-11 years old children and adults. They did not include 9 years old children in their study. Cepeda and colleagues [3] studied task switching with two groups: 7-9 and 10-12 years old children. The coarse grouping of ages makes it more difficult for precisely tracking developmental changes for these executive processes. It has also been suggested that the major development in executive control processes takes place between 6-10 years of age [30,31]. Hence, we examined children in each age level between 6 and 11 years.

All our participants performed a task in which they had to respond to two different task rules: discriminate the value of a number presented on a computer screen or decide on numerosity (deciding how many numbers were present on the screen). This design allowed us to compare the switch costs (decrement in reaction time due to switching between tasks) for trials in which responses were repeated against trials in which responses were switched. In addition, it also helped us to explore the role of compatibility in the context of task switching. We have kept the CTI and ITI/RCI at 0 ms, as previous studies have indicated that manipulating these two variables did not affect the performance of the children aged 7-12 years. The main focus was to study age-related changes only in children aged 6-11 years. Both the cue and target remained on the screen until response, allowing flexible time for participants to prepare themselves and then to respond. This also enabled us to examine the failure-to-engage hypothesis. In addition to the switch costs, we analyzed reaction time (RT) distributions to examine closely the mechanisms underlying task switching.

Error signal/feedback was provided to indicate the occurrence of error in a given trial. Even after providing an external error signal, the processes related to correcting the error (post-error-slowing) would still happen, which was measured through PES. RT distributions were also computed for the post-error trials to closely examine the mechanisms underlying error processing.

## Methods

### Participants

A total of 180 children in the age range of 6-11 years (30 in each of the six age levels) participated in the study. All participants had normal or corrected-to-normal visual acuity. No participant had participated previously in a task switching experiment. Permission was taken from the school's principal and teachers. Informed consent was obtained from the parents.

### Stimuli and apparatus

**Figure 1:**
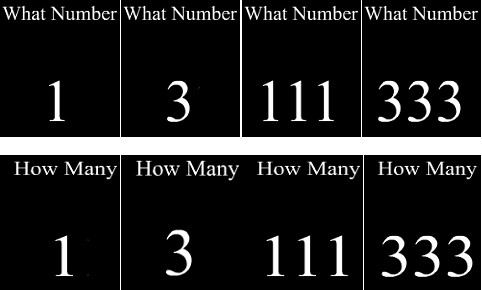
**Example of the stimuli used in the study**.

Four stimuli, either a single digit (1 or 3) or three digits (111 or 333) were presented at the center of a laptop computer screen. Above the target stimulus, the words "What Number" or the words "How Many" appeared depending on the task to be performed in that trial. The size of a digit was 1.43° × 0.956° (Figure 1). Participants sat at a distance of 60 cm from the laptop screen. Responses were made using the 1 and 3 keys on the numeric keypad. A commercially available research software, DirectRT (Empirisoft corporation, USA) was used for stimulus presentation and data collection.

### Procedure

All the children were tested individually in a quiet, dimly lit room at their respective schools. Participants were required to switch their attention between two different tasks: identification of a digit or counting the number of digits. Stimuli remained on the screen until response. Feedback (100 Hz tone) was given whenever participants made an error. A practice session with 75 trials preceded the experimental session consisting of 200 trials. In each trial, the task to be performed appeared along with the stimuli. The two tasks were "what number", in which the participant was instructed to identify the digit(s) and "how many", in which the participant was instructed to count the number of digit(s). Cue (task specification) and the target appeared simultaneously and the next trial started immediately after the response. The participant was asked to press "1" if the answer for either of the tasks was "1" and "3" if the answer for either of the tasks was "3". This resulted in eight different trial types based on the relationship between two consecutive trials in terms of task, response, and S-R compatibility. RT distributions were computed using the cumulative distribution function (CDF) of MATLAB. The differences in fast and slow trials were evaluated by computing the 5^th ^percentile and 95^th ^percentile values respectively for the overall task switch and task non-switch trials, the response switch and response non-switch trials, and the S-R compatible and S-R incompatible trials.

## Results

### Task switching

#### Reaction time

Analysis was performed with RTs for all the conditions for all the participants across age. RTs were submitted to a mixed ANOVA with age (6, 7, 8, 9, 10, 11) as a between subject variable and task type (task switch, task non-switch), response type (response-repetition, response-switch), and S-R compatibility type (S-R compatible, S-R incompatible) as within subject variables. There was a significant main effect for age, *F*(5, 174) = 59.7, *p *= .001; task type, *F*(1, 174) = 191.8, *p *= .001; response type, *F*(1, 174) = 53.9, *p *= .001; and S-R compatibility type, *F*(1, 174) = 64.3, *p *< .001. We performed post-hoc analysis with Bonferroni corrections. There was a decrease in RTs with increase in age. Overall RTs decreased significantly from the 9 to 10, *t*(58) = 9.26, *p *= 0.0001, and the 10 to 11, *t*(58) = 6.26, *p *= 0.0001, age groups. RTs were higher for the task-switch, response-switch, and S-R incompatible trials compared to the task-non-switch, response-repetition, and S-R compatible trials respectively. RTs were higher in the task-switch condition compared to the task-non-switch condition, indicating the presence of switch costs. RTs for response-switch were higher than response-repetition, indicating that response-switch slowed down responses. Similarly, RTs for the S-R incompatible trials was higher than the S-R compatible trials, indicating that S-R incompatibility also slowed the responses.

Age interacted with task type, *F*(5, 174) = 7.73, *p *= .001, response type, *F*(5, 174) = 5.12, *p *= .001, and S-R compatibility type, *F*(5, 174) = 2.91, *p *= .01. Post-hoc analysis for all the three interactions showed significant differences for the 6, 7, 8, and 9 years old children and no significant difference for the 10 and 11 years old children. RTs were significantly larger in the task-switch compared to the task-non-switch, the response-switch compared to the response-repetition, and the S-R incompatible compared to the S-R compatible trials for the 6, 7, 8 and 9 years old children. The results indicate the presence of switch costs in the 6 to 9 years old children. In addition, changing a response in two consecutive trials, or the presence of S-R incompatibility, resulted in the slowing of responses among the 6 to 9 years old children.

Task type interacted with response type, *F*(1, 174) = 156.5, *p *= .001. RTs were significantly less for the response-repetition as compared to the response-switch condition, when the task was repeated, *t*(29) = 18.2, *p *= 0.0001, indicating the presence of the repetition effect. However, RTs were higher for the response-repetition as compared to the response-switch condition, *t*(29) = 6.79, *p *= 0.0001, when task was switched, which indicated the presence of the reversed-repetition effect. These results indicate that response-switch slows down the response only when a task is not switched. Response-switch does not slow down the responses when a task is switched but may actually benefit responses. RTs were significantly faster when the task as well as the response was repeated, compared to the other three conditions. The three-way interaction between task type, and response type was significant, *F*(5, 174) = 14.5, *p *= .001. This is due to the presence of switch cost only in the response-repetition condition for the 6-9 year old children. The interaction between task type and S-R compatibility type was significant, *F*(1, 174) = 6.13, *p *= .01. The compatibility effect in the switch trials (142.5 ms) was larger than that obtained in the non-switch trials (72.5 ms).

**Figure 2:**
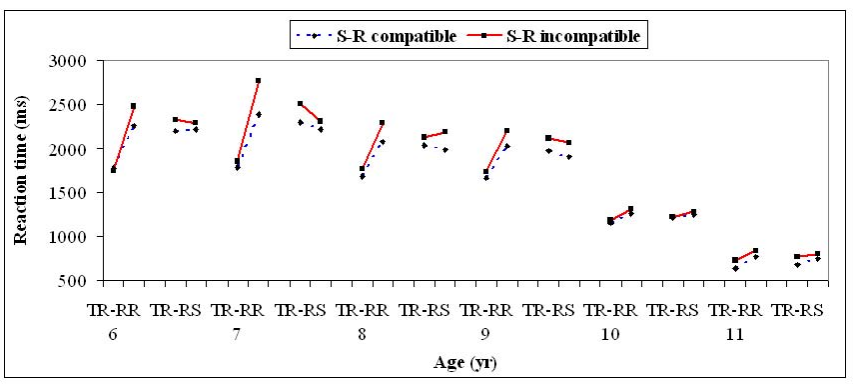
**Response latencies as a function of the task repetition/switch, the response repetition/switch, and the S-R compatibility/incompatibility for all the six age groups**. (TR = Task-Repetition; TS = Task-Switch; RR = Response-Repetition; RS = Response-Switch).

The interaction between task type, response type, and S-R compatibility type was significant, *F*(1, 174) = 10.3, *p *= .01. The compatibility effect was also significantly larger in the response-repetition, task-switch trials (184.5 ms) compared to the response-repetition, task-non-switch trials (63.2 ms). There was no significant difference in the compatibility effect obtained in the response-switch, task-switch trials (100.0 ms) compared to the response-switch, task-non-switch trials (112.0 ms). The four-way interaction among age, task type, response type, and S-R compatibility type was significant, *F*(5, 174) = 2.89, *p *= .01. The interaction between response-repetition, task type, and S-R compatibility was primarily present among children aged 6-9 years. A significant compatibility effect was obtained with response-repetition, task-switch trials with children aged 6 to 9 years (Figure 2).

We also obtained RT distributions using CDF for the task switch and task non-switch trials, the response switch and response repetition trials, the S-R compatible and S-R incompatible trials for each participant in each age level. RTs at the 5^th ^percentile (fast trials) and 95^th ^percentile (slow trials) were calculated for each participant. An ANOVA was performed between age and task type, age and response type, age and S-R compatibility type for the 5^th ^percentile and 95^th ^percentile values separately.

**Figure 3:**
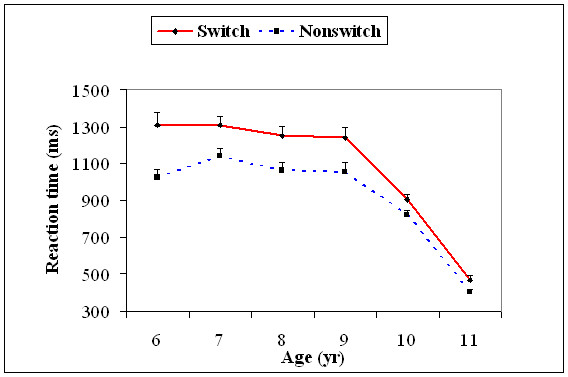
**Mean reaction time (+1SEM) of the 5^th ^percentile (fast trials) for the task switch and task non-switch conditions for all the six age groups**.

With the 5^th ^percentile value, there was a significant main effect for age, *F*(5, 174) = 54.2, *p *= .001, and task type, *F*(1, 174) = 164.3, *p *= .001. Age significantly interacted with task type, *F*(5, 174) = 6.67, *p *= .001. The 5^th ^percentile RTs on the switch trials were significantly slower than that for the non-switch trials for age 6, *t*(29) = 13.0, *p *= 0.0001, age 7, *t*(29) = 7.63, *p *= 0.0001, age 8, *t*(29) = 8.59, *p *= 0.0001, and age 9 years, *t*(29) = 8.42, *p *= 0.0001, and were similar for ages 10 and 11 years. The 5^th ^percentile RTs decreased significantly from 9 to 10, *t*(58) = 15.1, *p *= 0.0001, and 10 to 11, *t*(58) = 20.0, *p *= 0.0001, age groups on the switch trials. Similarly, it also decreased from 9 to 10, *t*(58) = 10.5, *p *= 0.0001, and 10 to 11, *t*(58) = 19.2, *p *= 0.0001, age groups on the non-switch trials. For the rest of the age groups, there was no significant difference in the response-repetition trials (Figure 3).

**Figure 4:**
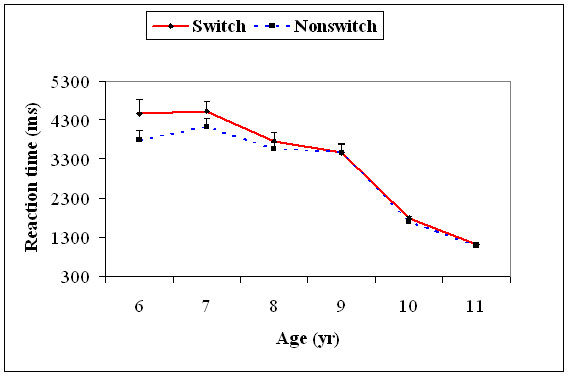
**Mean reaction time (+1SEM) of the 95^th ^percentile (slow trials) for the task switch and task non-switch conditions for all the six age groups**.

With the 95^th ^percentile value (characterizing slow trials), there was a significant main effect for age, *F*(5, 174) = 49.1, *p *= .001, and task type, *F*(1, 174) = 18,5, *p *= .001. Age significantly interacted with task type, *F*(5, 174) = 3.86, *p *= .01. The 95^th ^percentile value RTs of the switch trials (all age levels) were not significantly different from RTs on non-switch trials, except for age 6, *t*(29) = 7.18, *p *= 0.0001, and age 7, *t*(29) = 4.31, *p *= 0.01, years. The 95^th ^percentile RTs decreased significantly from 7 to 8, *t*(58) = 8.19, *p *< = 0.0001, 9 to 10, *t*(58) = 17.6, *p *= 0.0001, and 10 to 11, *t*(58) = 6.97, *p *= 0.0001, years among the switch trials. Similarly, the 95^th ^percentile RTs decreased significantly from 7 to 8, *t*(58) = 6.04, *p *= 0.0001, 9 to 10, *t*(58) = 18.3, *p *= 0.0001, and 10 to 11, *t*(58) = 6.53, *p *= 0.0001, years among the non-switch trials (Figure 4).

**Figure 5:**
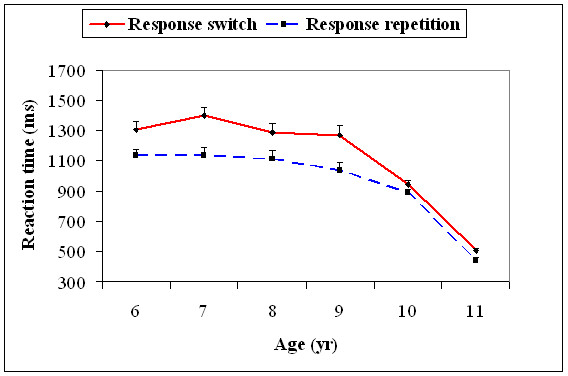
**Mean reaction time (+1SEM) of the 5^th ^percentile (fast trials) for the response switch and response repetition conditions for all the six age groups**.

With the 5^th ^percentile value, there was a significant main effect for age, *F*(5, 174) = 76.4, *p *= .001, and response type, *F*(1, 174) = 30.5, *p *= .001. Age significantly interacted with response type, *F*(5, 174) = 13.2, *p *= .001. The 5^th ^percentile RTs decreased significantly from 9 to 10, *t*(58) = 7.29, *p *= 0.0001, and 10 to 11, *t*(58) = 9.51, *p *= 0.0001, age groups on the response switch trials. On the response repetition trials, RTs significantly decreased only from 10 to 11, *t*(58) = 9.78, *p *= 0.0001, years of age. For the rest of the age groups, there was no significant difference in the S-R compatible trials (Figure 5).

**Figure 6:**
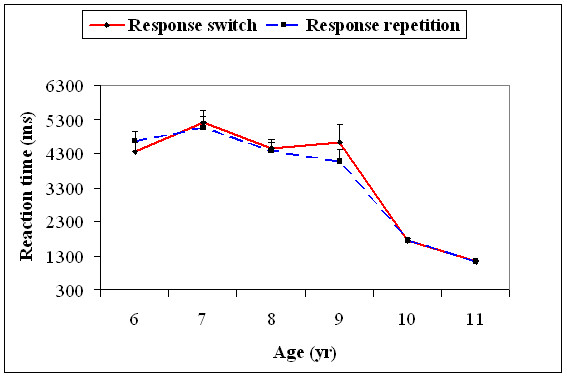
**Mean reaction time (+1SEM) of the 95^th ^percentile (slow trials) for the response switch and response repetition conditions for all the six age groups**.

With the 95^th ^percentile value (characterizing slow trials), there was a significant main effect for age, *F*(5, 174) = 57.8, *p *= .01. Age significantly interacted with response type, *F*(5, 174) = 2.67, *p *= .02. The 95^th ^percentile RTs decreased significantly only from 9 to 10 years for both the response switch, *t*(58) = 11.2, *p *= 0.0001, and the response repetition trials, *t*(58) = 9.06, *p *= 0.0001 (Figure 6).

**Figure 7:**
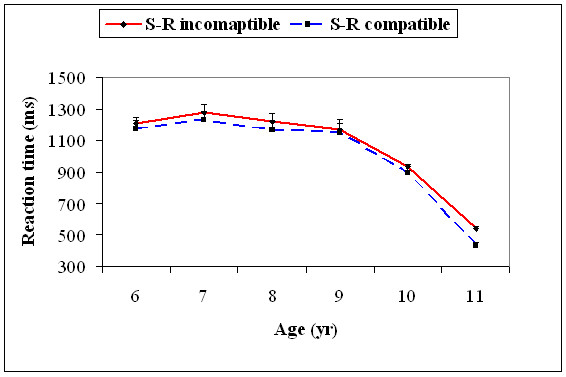
**Mean reaction time (+1SEM) of the 5^th ^percentile (fast trials) for the S-R compatible and S-R incompatible trials for all the six age groups**.

With the 5^th ^percentile value, there was a significant main effect for age, *F*(5, 174) = 63.2, *p *= .001, and S-R compatibility type, *F*(1, 174) = 32.4, *p *= .001. Age significantly interacted with S-R compatibility type, *F*(5, 174) = 16.7, *p *= .001. The 5^th ^percentile RTs decreased significantly from 9 to 10, *t*(58) = 5.90, *p *= 0.0005, and 10 to 11, *t*(58) = 9.69, *p *= 0.0001, age groups on the S-R incompatible trials. Similarly, on the S-R compatible trials it significantly decreased only from 9 to 10, *t*(58) = 6.31, *p *= 0.0001, and 10 to 11, *t*(58) = 11.4, *p *= 0.0001, years of age. For the rest of the age groups, there was no significant difference (Figure 7).

**Figure 8:**
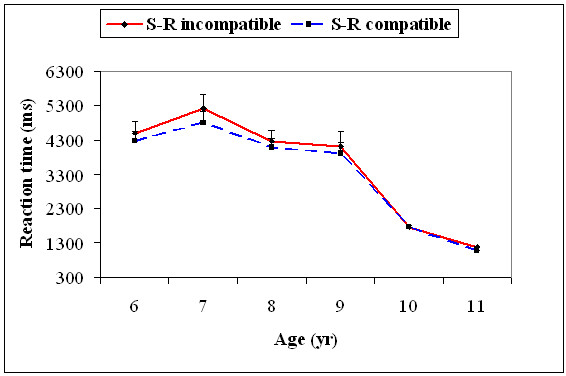
**Mean reaction time (+1SEM) of the 95^th ^percentile (slow trials) for the S-R compatible and S-R incompatible trials for all the six age groups**.

With the 95^th ^percentile value, there was a significant main effect for age, *F*(5, 174) = 43.6, *p *= .001. Age significantly interacted with the S-R compatibility type, *F*(5, 174) = 3.55, *p *= .004. The 95^th ^percentile RTs decreased significantly only from 7 to 8, *t*(58) = 4.31, *p *= 0.02, and 9 to 10 years, *t*(58) = 10.6, *p *= 0.0001, for the S-R incompatible trials, while it was only significant for 9 to 10 years, *t*(58) = 9.65, *p *= 0.0001, for the S-R compatible trials (Figure 8).

#### Switch costs

Trials with excessively short RTs (<100 ms) and error trials were excluded from the analysis. Switch costs (SC) were computed as a function of response (response-repetition and response-switch) and S-R compatibility (compatible and incompatible) for all the participants. A 6 × [2 × 2] mixed ANOVA with age (age levels: 6, 7, 8, 9, 10, and 11) as a between subject factor and response type (response-switch and response-repetition condition) and S-R compatibility type (S-R compatible vs S-R incompatible) as the within subject factors was performed. There was a significant main effect for age, *F*(5, 174) = 7.73, *p *= .001, response type, *F*(1, 174) = 156.5, *p *= .001, and S-R compatibility type, *F*(1, 174) = 6.13, *p *= .01. Overall SC decreased with an increase in age, with decrement in SC observed between 7 to 10 years of age. Switch costs was observed only with the response-repetition trials and not with the response-switch trials. Switch costs was also larger in the S-R incompatible trials compared to the S-R compatible trials.

Interaction between age and response type was significant, *F*(5, 174) = 14.5, *p *= .001. Significant SC was obtained for the response-repetition trials, compared to the response-switch trials, only for the 6, 7, 8 and 9 years old children. SC for the response-repetition trials decreased from 7 to 8 years, *t*(58) = 5.01, *p *= 0.001, and 9 to 10 years, *t*(58) = 4.83, *p *= 0.001. There was no significant difference in SC in the response-switch condition across all the age groups.

**Figure 9:**
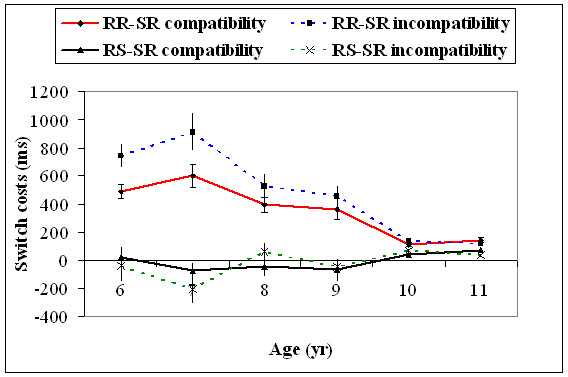
**Switch costs (+1SEM) as a function of the response repetition/switch and the S-R compatibility/incompatibility for all the six age groups**. RR = Response-Repetition; RS = Response-Switch; SR = Stimulus-Response

Two-way interaction between response type and S-R compatibility type, *F*(1, 174) = 10.3, *p *= .01, and the three-way interaction among age, response type, and S-R compatibility type was significant, *F*(5, 174) = 2.89, *p *= .01. SC was significantly more in the S-R incompatible condition, compared to the S-R compatible condition, when responses were repeated, *t*(29) = 5.87, *p *= 0.0001. However, it was only found for the age groups of 6, *t*(29) = 4.60, *p *= 0.01, and 7, *t*(29) = 5.69, *p *= 0.0001. For the rest of the age groups, it was not significant. In addition, SC significantly decreased from 7 to 8, *t*(58) = 7.10, *p *= 0.0001, and 9 to 10, *t*(58) = 5.92, *p *= 0.0001, age groups, with the S-R incompatible trials. For the S-R compatible trials, SC significantly decreased only between 9 to 10 years, *t*(58) = 4.59, *p *= 0.01. There was no difference in SC between the S-R compatible and S-R incompatible conditions when responses were switched. There was also a significant difference in SC between 7 to 8 years, *t*(58) = 4.81, *p *= 0.001, for the S-R incompatible, response-switch trials (Figure 9).

#### Post-error-slowing

**Figure 10:**
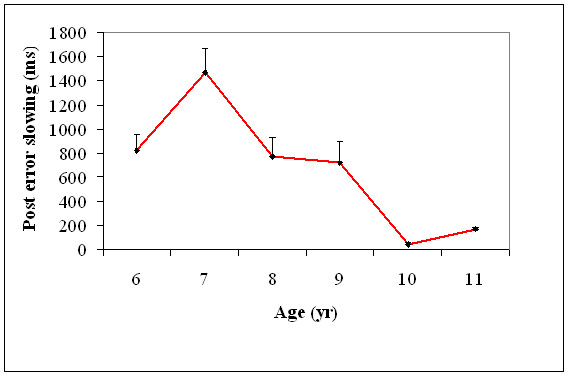
**Post error slowing (+1SEM) of all the six age groups**.

PES (a measure of error processing) was calculated as: [the mean RT from the correct trials immediately following an error] minus [mean RT from correct trials], for each participant. A one variable ANOVA was performed on PES values with age (6, 7, 8, 9, 10, and 11) as a between-subject variable. There was a significant main effect for age, *F*(5, 174) = 14.6, *p *= .001, indicating that PES changed with age. Post-hoc comparisons showed that PES increased between 6 to 7 years of age, *t*(58) = 4.81, *p *= 0.001, and then decreased from 7 to 8, *t*(58) = 5.21, *p *= 0.001, and 9 to 10, *t*(58) = 5.04, *p *= 0.001, years of age. PES did not decrease between 8 and 9 as well as 10 and 11 years of age (Figure 10).

**Figure 11:**
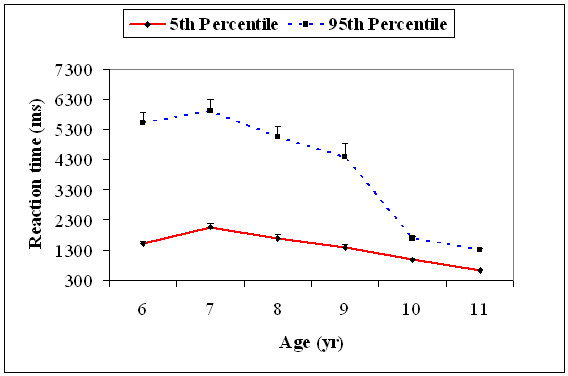
**Mean reaction time (+1SEM) of the 5^th ^(fast trials) and the 95^th ^percentile (slow trials) for correct trials immediately following an error for all the six age groups**.

Reaction time distributions were obtained for the RTs of those correct trials immediately following an error, and we also computed the RT values at 5^th ^percentile and 95^th ^percentile as a measure of fast and slow trials respectively for all the participants. A one variable ANOVA was performed with age (6, 7, 8, 9, 10, and 11) with the 5^th ^and the 95^th ^percentile RT values separately. There was a significant main effect for age for the 5^th ^percentile, *F*(5, 174) = 24.5, *p *= .001 and 95^th ^percentile, *F*(5, 174) = 42.9, *p *= .001, RT values. Post-hoc comparisons indicated that the RT values at the 5^th ^percentile increased from 6 to 7, *t*(58) = 5.32, *p *= 0.001, and then decreased from 7 to 8, *t*(58) = 3.66, *p *= 0.05, and 9 to 10, *t*(58) = 4.02, *p *= 0.01, years of age indicating changes in the fast trials. However, the 95^th ^percentile RT values decreased only from 9 to 10 years of age, *t*(58) = 8.86, *p *= 0.0001, indicating changes in the slow trials only between ages 9 and 10 (Figure 11).

Results indicated that the developmental pattern of PES was similar with respect to the switch cost response repetition condition. Therefore, to further confirm the association between PES and switch cost of response repetition, correlation analyses was performed between PES and switch cost of response repetition, as well as PES and switch cost of response switch condition. A significant positive correlation was observed between PES and switch cost of response repetition condition, *r *= 0.454, *p *< .001. There was no significant correlation between PES and switch cost of response switch condition. Previous research has been linked the switch cost of the response repetition condition with the inhibitory processes [14,15], indicating inhibition could be the common mechanism between task switching and error processing.

## Discussion

We examined from a developmental perspective whether the two control processes, task switching and error processing share common underlying mechanisms or whether these are completely dissociable processes. We hypothesized that if the mechanisms underlying task switching and error processing are completely dissociable, then their developmental trends would be different. Our results suggest that inhibition could be a common mechanism underlying task switching and error processing. Failure to maintain task set could be a different mechanism which underlies only in task switching but not in error processing. Thus, with respect to the failure to maintain task set task switching and error processing are dissociable processes.

Previous studies have examined the development of task switching by employing a small number of age groups among children. For example, Crone and colleagues [4] used two age groups (7-8 and 10-12 years) and Cepeda and colleagues [3] used two age groups (7-9 and 10-12 years) for studying task switching performance in children. In comparison, we have examined six age levels (6-11 years) to accurately measure the developmental patterns in task switching among children. This has enabled us to investigate the differences in performance across 6 to 11 years of age.

Our results show that the overall switch costs reduced from 7 to 10 years of age, indicating that executive control processes develop significantly during this period. Consistent with our results, developmental studies on inhibitory control have shown a significant development between 7.5 to 9.5 years of age, followed by 9.6 to 11.5 years [32]. When we look at the overall switch costs, it appears that task switching is characterized by gradual development. However, a closer look at switch costs as a function of response-repetition and S-R compatibility revealed that the development of task switching was not gradual.

The reduction in switch costs varied as a function of response repetition/switching [4,33,34]. Similar to Crone and colleagues [4], we observed switch costs when the responses were repeated (reversed-repetition effect), and this effect decreased with age. More importantly, we found that the most striking developmental advances in the reversed-repetition effect occurred between 7 to 8 and 9 to 10 years of age, followed by stabilization. Children benefited from repeating the same responses in task repetition trials (repetition benefit). One possibility is that children experience greater carry over effects from the previously activated S-R association, because the binding between stimuli and responses is stronger in children. This interpretation is consistent with the results from previous developmental studies [4,35,36], which showed that when trials occur in rapid succession, the stimuli are processed automatically, resulting in performance benefits. Following this interpretation, children may adjust associations between responses and tasks, resulting in benefits with task repetition, but raising costs with task switching [33]. This is the advantage with repetition.

In our study, whether it is a task or response switch, children with 6 to 10 years of age found it difficult to make a change. Crone and colleagues [4] showed a pronounced reaction time slowing on task-switch trials with response-repetition among 7-8 years old and 10-12 years old children. They also interpreted this finding (of switch costs in task-switch trials with response-repetition) in terms of carry-over effects from the previous S-R association. We did not get any significant difference in reaction time between task-repetition and task-switch conditions when responses were switched for all the age groups, while there was a significant difference between task-repetition and task-switch conditions when responses were repeated. This indicates that there was an advantage for task as well as response repetition. This is consistent with the finding from the Crone and colleagues [4] study. However, unlike our study, they got significant difference in reaction time between the task-repetition and task-switch conditions only when responses were switched.

There were some differences in results obtained in our study and the Crone et al., study [4] study. For example, we did not observe switch costs when the responses were switched. However, Crone and colleagues [4] did find switch costs when responses were switched (although reduced compared to response-repetition condition). We also found larger switch costs in the S-R incompatible condition as compared to the S-R compatible condition. However, Crone and colleagues [4] reported that the switch costs were larger when switching to the compatible task than to the incompatible task, and this effect did not differ between the age groups. Interestingly, unlike Crone and colleagues [4], we observed that age interacted with the response type (repetition/switching) and S-R compatibility type (S-R compatibility/incompatibility). For repeated responses, major development occurred in SC between 7 to 8 and 9 to 10 years of age, for both the compatible and incompatible conditions. The effect was magnified in the incompatible condition compared to the compatible condition.

Some of the differences between the findings from our study and other similar studies [3,4] could be attributed to differences in stimuli and task conditions. The current study employed no cue-to-target and response-to-cue intervals, resulting in very little time to prepare for the next task. Crone and colleagues [4] manipulated RCI at three levels (50 ms, 500 ms and 1250 ms) and used a spatial task, rather than the identity or numerosity judgments used in the current study. In their study, the S-R compatibility was based on the location of the target and the response to be made, whereas in our study it was based on the identity of the stimuli and the response. The shape or color targets redundantly cued compatible or incompatible responses. Crone and colleagues [4] used response-stimulus interval of 50 ms and the task was specified by the stimuli themselves. In our study, a separate cue specified the task (set of stimuli were the same for both the tasks), which was similar to Cepeda and colleagues [3] study. There were differences between our and the Cepeda and colleagues [3] study as well. They [3] varied RTI and CTI, with the smallest RTI and CTI of 100 ms each, which was the closest values to the null RTI and CTIs we used in our study. While Cepeda and colleagues [3] used similar stimuli and tasks, they did not examine the critical effects of response-repetition or response-switch.

The present results suggest that the major development in overall switch costs takes place between 7 to 10 years of age. However, a closer look at RT distributions for overall task switch and task non-switch trials with the 5^th ^percentile and 95^th ^percentile values indicate that the decrement in switch costs may be due to the overall speeding up of the fast trials in 9 to 11 years of age, and speeding up of the slow trials in 7 to 8 and 9 to 11 years of age, in both the task switch and task non-switch trials. These results indicate that the failure to maintain task set in task switch and task non-switch trials improves between 9 and 11 years of age.

Our results indicate that the major development in error processing as measured by PES takes place between 6 to 10 years of age, with an initial increase in PES, followed by a decrease. The decrease from 7 to 10 years is not uniform, with some decrease between the ages 7 and 8, followed by a substantial reduction in PES between the ages 9 and 10. A closer look at the RT distributions for those trials immediately following an error reveals that the largest decrease, occurring between 9 and 10 years, is primarily due to decrease in RTs of the 95^th ^percentile value, indicating a speeding up of the RTs of the slow trials. This indicates that the occasional inability to recover from prior error trials (which result in the larger number of slow trials) gets reduced with an enhanced ability to respond appropriately in the subsequent trial between 9 to 10 years of age. Our results are supported by the ERP correlates of error processing [5]. Davis and colleagues [5] reported that ERN amplitude in error trials increased with age. However, the ERN was very small in most young children (ages 7 to 12 years of age), which indicates that unconscious detection of errors is less developed in this age cohort. Even in the absence of the ERN, they all produced a robust *Pe*, as did the adults, which indicates that children with 7 to 12 years of age are able to consciously recognize the errors and are able to adjust their performance after an error.

So far, no study has closely examined the development of error processing using PES in children between 6 to 11 years, which is a period of major development of executive function in children. A few ERP studies have briefly discussed the behavioral component (PES) of error processing [6,5,37]. For example, Wiersema and colleagues [6] examined the developmental trajectory of error processing in children (aged 7-8), young adolescent (13-14), and adults (age 23-24). They found no difference between age groups with respect to PES. Davis and colleagues [5] also reported no difference between age groups for PES. In contrast, Hogan and colleagues [37] observed an increase in the amount of PES from adolescence (age 12-19) to adulthood (age 19-22). There could be two possible accounts for the diverging results. First, all these studies have combined children with different ages, which makes it difficult to precisely track the developmental changes in error processing. Secondly, the different results can at least partly be attributed to differing task requirements. For example, Wiersema and colleagues [6] used a Go/No-Go task, unlike the task switching paradigm used in the present study. Hogan and colleagues [37] used forced-choice response tasks of varying complexity and the difference was only found in the most complex task, indicating that task complexity may play a critical role in highlighting the developmental trends in error processing.

Major development in task switching takes place between 7 to 10 years of age, while major development in error processing takes place between 6 to 8 and 9 to 10 years of age. The development of task switching as a function of response repetition/switching - SR compatibility/incompatibility is similar to the developmental trend of PES. With respect to the switch cost in the response repetition condition (reversed-repetition effect), the developmental pattern of PES was similar to that of task switching. Since reversed-repetition effect has been linked to the inhibitory process [13,14], inhibition could be a common mechanism underlying task switching and PES.

In addition to inhibition, other mechanisms underlie task switching and PES. For example, cue encoding may play a critical role in task switching [38] and orienting to the error tone may underlie PES (Burns JT: The effects of error on reaction time in a serial reaction task, unpublished). In the present study, the CTI was 0 ms (cue and the target both appeared simultaneously), so participants were not required to remember the cue regarding the task to be performed. Orienting accounts of PES suggest that PES is caused by the relative infrequency of errors which cause attentional capture. However, the orienting account of the PES is also linked to orienting to the inhibitory process. For example, it has been suggested that the occurrence of an error was followed by an orienting response which inhibited rather than facilitated subsequent responses (Burns JT: The effects of error on reaction time in a serial reaction task, unpublished). In line with this, Barcelo and colleagues [39] reported slowing after infrequent events (oddballs) to the task. Hence, the orienting account also suggests an inhibitory mechanism explanation for PES, which further strengthens our argument that inhibition could be a common mechanism underlying task switching and error processing.

In addition to common mechanism (inhibition) between task switching and error processing, failure to maintain task set could be a different mechanism which underlies only in task switching but not in error processing. Thus, with respect to the failure to maintain task set task switching and error processing are dissociable processes. A closer look at the RT distributions of task switch and task non-switch, response switch and response repetition, S-R compatible and S-R incompatible trials, and post error trials reveal that the development of both the processes (task switching and error processing) differed in terms of difference in age-related changes observed in the 5^th ^and 95^th ^percentile value. For example, major reduction in switch costs was observed between 9 to 11 years, primarily due to the sudden decrease in the 95^th ^percentile value, indicating the decrement in RTs in the slow trials. Major reduction in values was obtained for RT distribution of trials immediately following an error in the 5^th ^and 95^th ^percentile. This indicates a decrease in RTs in both the fast and slow trials respectively. It appears that the failure to maintain a task set occasionally on task switch and task non-switch trials stayed constant in children with 6 to 9 years of age. However, these failures come down between 9 and 11 years of age.

In addition, major changes in RT occurred between 9 to 11 years, in both the fast and slow trials of task type (task switch and task repetition), which was also observed with other two variables such as response type (response switch and response repetition), and S-R compatibility type (S-R compatible and S-R incompatible). These results indicate that the major age-related changes in task switching occurred in the later age group (9 to 11 years). However, in the PES, major development was observed in the early ages such as 6 to 9 years in fast trials and 9 to 10 years in slow trials. These results indicated that the major age-related changes in PES occurred in the early age group (6 to 9 years) as well as in the later age group (9 to 10 years). These results suggest that both task switching and error processing show some differences with respect to failure-to-maintain task set.

Together, these results throw some light on the long-running debate between unitary and component views of executive control. The unitary theory of executive control posits a unified mechanism or a common resource underlying various aspects of executive control, while component theories of executive control argued that executive control consists of a number of distinct but interacting components such as task switching and error processing. Results of the present study support component views of executive control. We also found that there are shared as well as different mechanisms underlying control processes such as task switching and error processing.

### Limitations

In the present study we have not manipulated the response-cue interval or the cue-target interval. These could be further manipulated in future studies to see the effect of these variables in the development of task switching in children.

## Conclusion

It appears that the major development in task switching takes place between 7 to 10 years of age, which indicates that task switching develops continuously. However, when we look at the switch costs as a function of response repetition/switching - SR compatibility/incompatibility then the development in task switching was not continuous. Major development in error processing takes place between 6 to 8 and 9 to 10 years of age, which indicates that the development of error processing is not continuous, but occurs in spurts. The developmental pattern of error processing is similar to the developmental pattern of task switching in response-repetition condition, in both the compatible and incompatible trials, indicating that inhibition could be a common mechanism underlying both the processes. However, with respect to the mechanism underlying failure-to-maintain task sets, the two processes of error processing and task switching are dissociable. The present study supports the component view of executive control. It also has implications for developmental disorders like ADHD characterized by executive control deficits.

## List of abbreviations

ADHD: Attention Deficit Hyperactivity Disorder; SC: Switch Costs; S-R: Stimulus-Response; PES: Post-Error-Slowing; ERP: Error Related Potential; ERN: Error Related Negativity; Pe: Error-Positivity; RT: Reaction Time; CDF: Cumulative Distribution Function; RCI: Response-Cue Interval; ITI: Inter-Trial Interval; CTI: Cue-Target Interval.

## Competing interests

The authors declare that they have no competing interests.

## Authors' contributions

RG designed, collected data, performed analysis, and wrote the manuscript. BRK helped in the design and writing of the manuscript. NS helped in design and data analysis, and wrote the manuscript. All authors have read and approved the final manuscript.
